# Socioeconomic Inequalities and Type 2 Diabetes Comorbidities: A Systematic Review and Meta‐Analysis on Observational Studies

**DOI:** 10.1002/edm2.70160

**Published:** 2026-01-22

**Authors:** Mansour Bahardoust, Sheida Shokohyar, Farzad Maleki, Atefe Shafiee, Farshid Monshizadeh Tehrani, Azin Ghaffari

**Affiliations:** ^1^ Department of Epidemiology School of Public Health & Safety, Shahid Beheshti University of Medical Sciences Tehran Iran; ^2^ Mass General Hospital Institute of Health Professions Boston Massachusetts USA; ^3^ Brigham and Women's Hospital, Harvard Medical School Boston Massachusetts USA; ^4^ Child and Adolescent Mental Health Center, Mental Health Services, Capital Region of Denmark Copenhagen Denmark; ^5^ Rajaie Cardiovascular Medical and Research Center, Iran University of Medical Sciences Tehran Iran

**Keywords:** comorbidities, socioeconomic inequalities, type 2 diabetes

## Abstract

**Background:**

Previous studies have reported inconsistent findings regarding the relationship between socioeconomic status (SES) inequalities and comorbidities in patients with diabetes mellitus (DM). This systematic review and meta‐analysis aims to evaluate, for the first time, the association between SES inequalities and comorbidities in individuals with DM.

**Methods:**

Two independent investigators searched the PubMed, Embase, Scopus, Web of Science and Google Scholar databases using MeSH terms to identify studies that investigated the association between SES and DM comorbidities up to 15 December 2025. This systematic review followed the PRISMA 2020 checklist. Heterogeneity among studies was assessed using Cochran's *Q* and *I* ^2^ statistics. Meta‐regression was used to control for heterogeneity; Egger's test assessed publication bias.

**Results:**

Thirteen studies involving 757,599 DM patients were included. A pooled estimate of 13 studies showed that low SES, compared with moderate or high SES, was significantly associated with an increased probability of DM comorbidities (OR: 1.43; 95% CI: 1.30, 1.59; *I* ^2^: 92.7, *p*: 0.01). Subgroup analysis of 12 studies showed that the probability of DM comorbidities was different in men (OR: 1.30; 95% CI: 1.28, 1.32) and women (OR: 1.39; 95% CI: 1.36, 1.42).

**Conclusion:**

The chance of developing type 2 diabetes comorbidities in patients with T2DM of low SES, especially in women, may be higher than in patients with middle and high SES. Improvements in healthcare systems and interventions to reduce inequalities in SES in patients with type 2 diabetes, especially in patients with low SES, are recommended.

## Graphical abstract

Graphical abstract

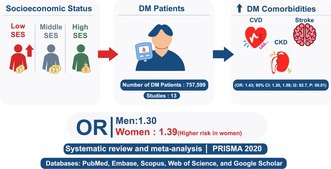

## 1 Introduction

Type 2 diabetes (T2DM) is recognised as a global epidemic in all populations, which is increasing significantly worldwide [1]. According to recent studies, more than 537 million people between the ages of 20 and 79 worldwide have diabetes, which is expected to increase by 46% by 2045 [2, 3]. People with low socioeconomic status (SES) are disproportionately affected by diabetes [4, 5]. Previous studies have demonstrated the association of SES inequalities with the incidence of T2DM, higher HbA1c levels, comorbidities development and T2DM complications [6, 7, 8, 9, 10, 11, 12, 13]. The substantial occurrence of T2DM and its related impact contribute significantly to developing complications and comorbidities [14, 15].

SES, determined by education, income and occupation, is a key predictor of diabetes mellitus (DM). Global disparities persist, with low SES groups experiencing greater disease burden despite advances in healthcare delivery and prevention [16, 17, 18, 19]. In addition to care services, SES disparities have been reported to be associated with diabetes care access and utilisation [20, 21]. Recent reviews have shown that the risk of DM comorbidities is higher in DM patients with low SES than in patients with higher SES, probably caused by problems following a healthy lifestyle [6, 8, 13]. In DM patients, the relationship between low SES and higher morbidity and mortality has been reported based on gender and period [6]. However, less evidence is available on the impact of low SES on increased DM comorbidities, and the results of reported studies in this area need to be more consistent. Several studies showed that low SES was associated with increased DM comorbidities [12, 22, 23]. While in several studies, it has been reported that low SES is not associated with DM comorbidities or has a predictive role [14, 24, 25]. However, the association of SES with DM comorbidities remains unclear.

In this systematic review and meta‐analysis, for the first time, we evaluated the relationship between SES and DM comorbidities in diabetic patients.

## 2 Methods

This study was based on the Preferred Reporting Items for Systematic Reviews and Meta‐Analyses (PRISMA) [26]. In this systematic review, based on the review of the literature, those suffering from at least one of the diseases of blood pressure, hyperlipidemia, thyroid disorders, heart failure, heart attack, angina pectoris, myocardial infarction, stroke, heart failure, retinopathy, nephropathy and polyneuropathy as DM comorbidities were defined. The primary studies classified SES inequalities into three groups: low, middle and high.

The definition of SES varied between studies; however, a combination of three factors—education, income and occupation—was considered as determinants of SES [27]. We considered the effect of different definitions of SES between studies on the overall outcome in meta‐regression analyses.

### 2.1 Methods for Literature Search

In this systematic review and meta‐analysis, after defining the research question by a team of epidemiologists, internists and health policymakers based on the PICO formula (population, interventions, comparators and outcomes), we searched PubMed/Medline, Scopus and Web of Science databases as well as study references to find related articles based on the search strategy defined for each database. The general search strategy was defined using mesh terms and the research question. Two independent researchers searched sources. The search was updated on 15 December 2025. The search for sources was conducted using the terms type 2 diabetes, socioeconomic inequalities and comorbidity. The general search strategy for searching sources is reported in the Supporting Information.

The PICO formula was defined as follows: (1) population: patients with T2DM, (2) exposure: socioeconomic inequalities (low socioeconomic level), (3) comparison: high or moderate socioeconomic level and (4) outcome: comorbidities.

### 2.2 Eligibility Criteria

The inclusion criteria for this meta‐analysis included the main observational studies (cohort, cross‐sectional and case–control) that investigated the relationship between SES inequality and DM comorbidities in T2DM, examining the relationship between SES inequality and DM comorbidities in general and based on subgroups of SES and English articles. Studies that examined the relationship between poor SES and type 1 diabetes comorbidities, case reports, letters to the editor, interventional studies, studies that used ethnic group as an index of SES, studies conducted on specific and ethnic groups, review and meta‐analysis articles, and lack of access to the full text of the article were defined as exclusion criteria.

### 2.3 Screening and Data Extraction

Two independent researchers initially searched the databases. Duplicate studies were identified and excluded between databases using EndNote version 22. Two independent researchers assessed the articles for relevance of titles, objectives and abstracts to the research question.

Data extraction was performed in Excel by two independent researchers using a designed checklist. The variables were determined based on the research question through a literature review and a panel of experts. The variables of this systematic review include the first author, the year and county of the study, the study design, total number of DM patients, the number of patients with DM comorbidities, the mean age of the patients, the SES class (low, middle, high), gender distribution, education level, income level, the effect size of the overall relationship between SES with DM comorbidity, the effect size of the SES relationship based on gender and income subgroups (odds ratio [OR] and 95% confidence interval [CI]). Two independent researchers (M.B.) and (A.G.) did the screening and data extraction. If there was a difference in the variable time, the third researcher resolved the difference.

### 2.4 Quality Assessment

The Newcastle‐Ottawa Scale (NOS) checklist for assessing the quality of nonrandomized studies in meta‐analysis was used to assess the quality of cross‐sectional studies included in this systematic review [28]. The quality of cohort studies was assessed with the Newcastle‐Ottawa Quality Assessment Form for Cohort Studies checklist [29]. The quality of studies based on these checklists is evaluated in three main sections: selection, comparability and result/introduction, and points are determined for each section (Table S1).

### 2.5 Statistical Analysis

Data were analysed using STATA version 17 statistical software. Meta‐prop command with 95% confidence interval (95% CI) was used to estimate the prevalence of DM comorbidities. The effect size of SES with DM comorbidity was reported by odds ratio (OR) at a 95% confidence interval (95% CI) by the Random‐effects method. To evaluate the degree of heterogeneity and inconsistency between studies, Cochran's *Q* and *I* ^2^ tests were used. Egger's test analysed publication bias for studies, and results were presented with funnel plots. DM comorbidities were analysed in gender and income subgroups—meta‐regression controlled heterogeneity between studies. Sensitivity analysis was used to estimate the effect size of each study on the overall result, and the weight of each study was determined.

## 3 Results

**FIGURE 1:**
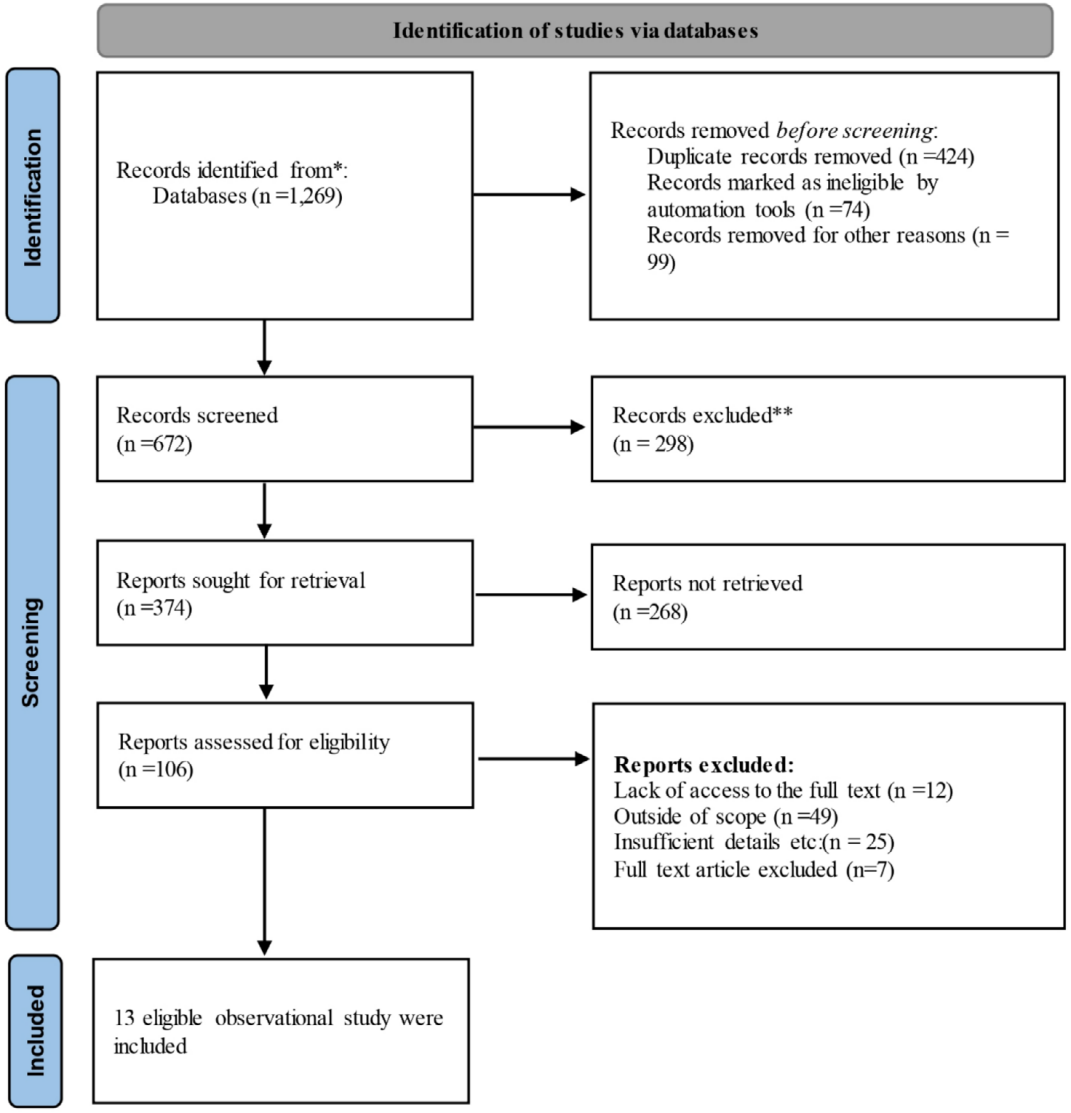
Flowchart page of studies based on PRISMA 2020.

In the initial search, 1269 primary articles were found. After initial screening of articles based on inclusion and exclusion criteria by two independent researchers, 374 articles remained. The full text of 106 studies was reviewed in full. Finally, 13 observational studies (6 cross‐sectional and 7 cohorts) [12, 14, 22, 23, 24, 25, 30, 31, 32, 33, 34, 35, 36], including 757,599 patients with type 2 diabetes, were included in this meta‐analysis (Figure 1).

The mean age of the patients in this study was 61.9 ± 3.3 years. 52.1% of patients were male. In terms of SES, most patients were at a middle level. Hypertension, hyperlipidemia and heart diseases were the most common diseases with diabetes, with 76% (95% CI: 65%, 87%), 69% (95% CI: 55%, 73%) and 49% (95% CI: 38%, 60%) respectively. The overall prevalence of microvascular complications in diabetic patients was 26.1% (19.1%, 33.3%). Most of the studies were conducted in developed countries. Almost 65% of patients had more than 10 years of education. The characteristics of the studies included in this meta‐analysis are reported separately in Table 1.

**TABLE 1:** Characteristics of patients in the studies and the quality of the included studies.

Author	Study design	Country	Sample size	Comorbidities	Age	Sex (male %)	SES level (%)	Education > 10 years	Quality of studies
R Dray‐Spira (2008) [30]	Cross‐sectional	USA	19,105	72.2%	60.4	47.9%	Low: 16.6% Middle: 23.7% High: 59.7%	66.6%	Moderate
V Fano (2013) [25]	Retrospective cohort	Italy	27,642	80.9%	67.5	50.5%	Low: 18.4% Middle: 57.5% High: 24.1%	NA	Good
PC Chen (2015) [31]	Retrospective cohort	Taiwan	57,791	68%	62.6	50.7%	Low: 49.3% Middle: 33.5% High: 17.2%	57.0%	Good
J Walker (2016) [23]	Prospective cohort	UK	126,648	80%	52.4	55.4%	Low: 25% Middle: 35% High: 40%	75.0%	Good
B Ibáñez (2018) [12]	Cross‐sectional	Spain	32,638	75%	66.1	55.7%	Low: 16.7% Middle: 57.3% High: 26%	72.1%	Good
N Ali (2019) [32]	Cross‐sectional	Bangladesh	825	41.1%	51.2	48.0%	Low: 25.1% Middle: 14.4% High: 60.5%	27.0%	Moderate
T Biswas (2019) [24]	Cross‐sectional	Bangladesh	1052	81.5%	54.1	42.8%	Low: 32.1% Middle: 12.9% High: 55%	37%	Good
L Bartolini (2020) [33]	Cross‐sectional	Italy	35,521	77%	60.2	52.6%	Low: 65.7% Middle: NA High: 364.3	69%	Good
P Jiang (2020) [34]	Retrospective cohort	Japan	58,349	94.7%	69.2	45.8%	Low: 29.4% Middle: 58.8% High: 11.8%	NA	Good
AC Falkentoft (2021) [35]	Retrospective cohort	Denmark	57,106	65%	59.4	55.2%	Low: 25% Middle: 25% High: 50%	81.9%	Good
S Kundu (2022) [36]	Prospective cohort	Bangladesh	1262	43%	60.2	51.8%	Low: 20.1% Middle: 48.4% High: 31.5%	62.0%	Moderate
B Safieddine (2023) [14]	Cross‐sectional	German	283,468	92.1%	54.1	47.1%	Low: 5% Middle: 84.4% High: 10.6%	55.5%	Good
J Uddin (2023) [22]	Cross‐sectional	Canada	56,192	73%	58.7	52.0%	Low: 33% Middle: 19% High: 48%	70.0%	Good

### 3.1 Prevalence of T2DM Comorbidities

**FIGURE 2:**
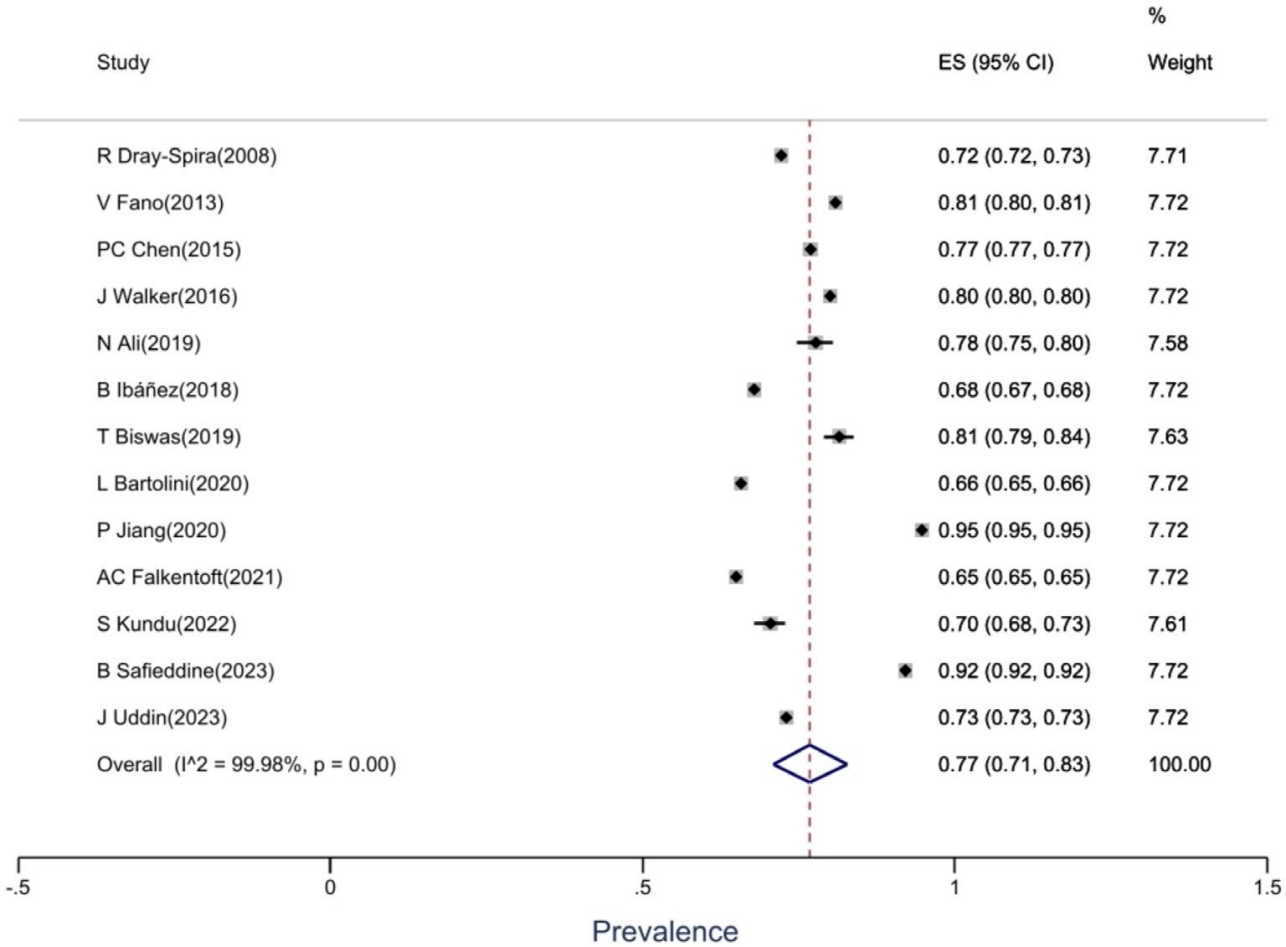
Forest plot of the prevalence of DM comorbidities.

The pooled prevalence of T2DM comorbidities was 77% (95% CI: 71%, 83%). In other words, out of every 100 patients with DM, 77 patients had at least one other comorbidity at the same time (Figure 2).

### 3.2 SES Inequalities and DM Comorbidities

**FIGURE 3:**
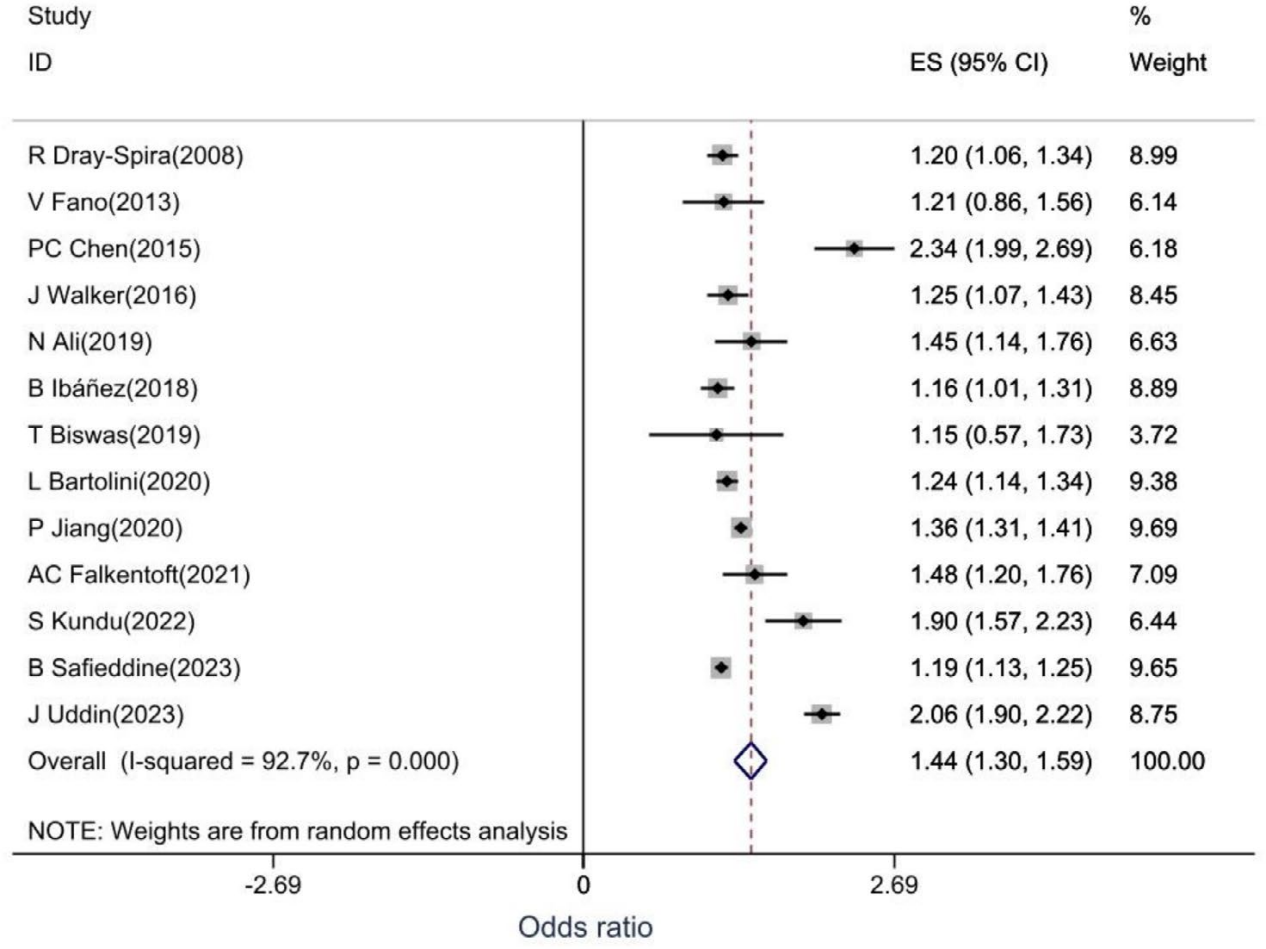
Relationship between DM comorbidity and SES.

The relationship between moderate and high SES compared to low with DM comorbidities was investigated in 13 studies [12, 14, 22, 23, 24, 25, 30, 31, 32, 33, 34, 35, 36]. The pooled estimation showed that low SES compared to middle or high SES level was significantly related to the increased probability of comorbidities (OR: 1.43; 95% CI: 1.30, 1.59; *I* ^2^: 92.7, *p*: 0.01) (Figure 3). Due to the high heterogeneity between the studies, adjusted meta‐regression was performed based on the quality of the studies. In line with the primary results, low SES was significantly associated with an increased probability of DM comorbidities (OR: 1.35; 95% CI: 1.25, 1.46; *I* ^2^: 15.7, *p*: 0.01).

### 3.3 Subgroup Analysis

**FIGURE 4:**
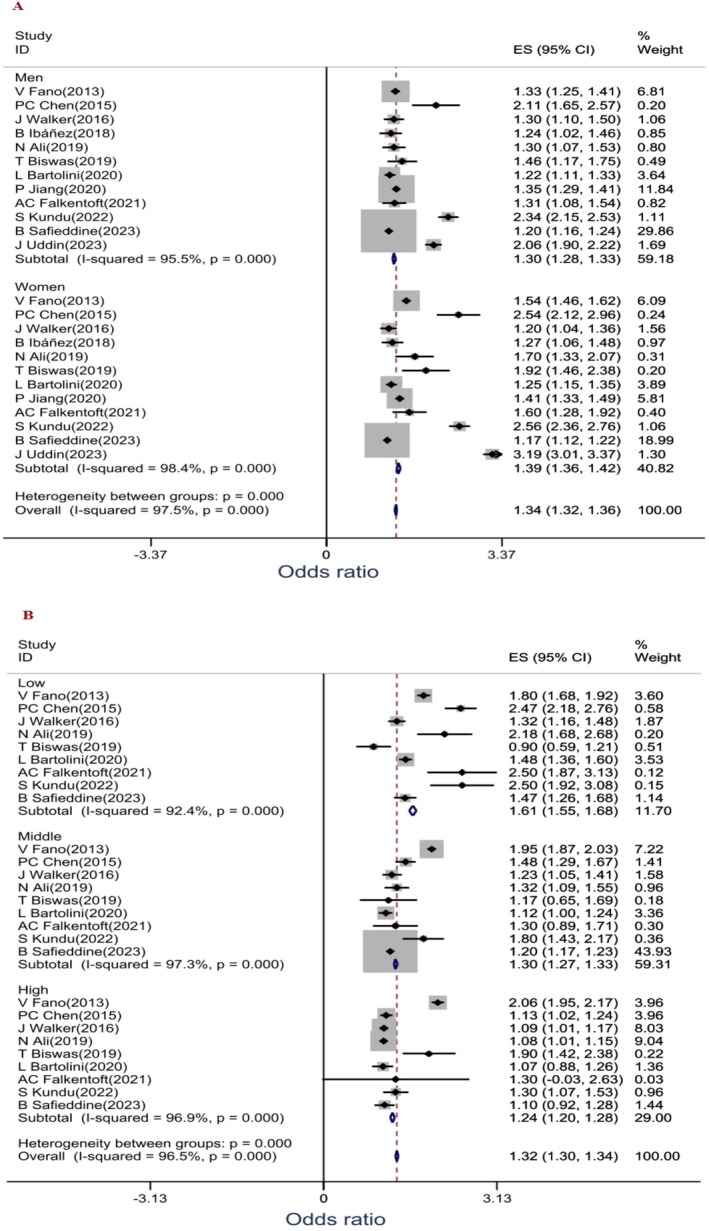
The relationship between DM comorbidity and SES based on gender (Forrest plot A) and income level (Forrest plot B) of diabetic patients.

The relationship between SES inequalities and DM comorbidities, separated by sex and income subgroups (low, middle and high), was investigated in 12 [12, 14, 22, 23, 24, 25, 31, 32, 33, 34, 35, 36] and 9 [14, 23, 24, 25, 31, 32, 33, 35, 36] studies, respectively. The subgroup analysis of 12 studies showed that the probability of DM comorbidities differed in men and women (Figure 4, Forest plot B). Subgroup analysis by income level showed that the probability of DM comorbidities was lower in patients with high levels compared to medium and low (Figure 4, Forest plot A).

### 3.4 Sensitivity Analysis and Meta‐Regression

A sensitivity analysis was performed based on each study's results, and each study's effect on the overall estimate was determined (Figure S1). The results of the meta‐regression showed that the comorbidity rate (per 10%), mean age, education years, quality of studies, sample size and geographic region were significantly associated with the overall effect (*p* < 0.05) (Table 2).

**TABLE 2:** Meta‐regression model of the effect of variables on effect size.

Variable	*β*	SE	*p*
Comorbidity rate (per 10%)	0.24	0.11	0.004
Education > 10 years	−0.41	0.19	0.028
Mean age (year)	0.21	0.09	0.001
Quality of studies (good vs. < good)	0.35	0.15	0.03
Sample size	0.87	0.21	0.001
Geographical region	−0.22	0.11	0.008

### 3.5 Publication Bias

**FIGURE 5:**
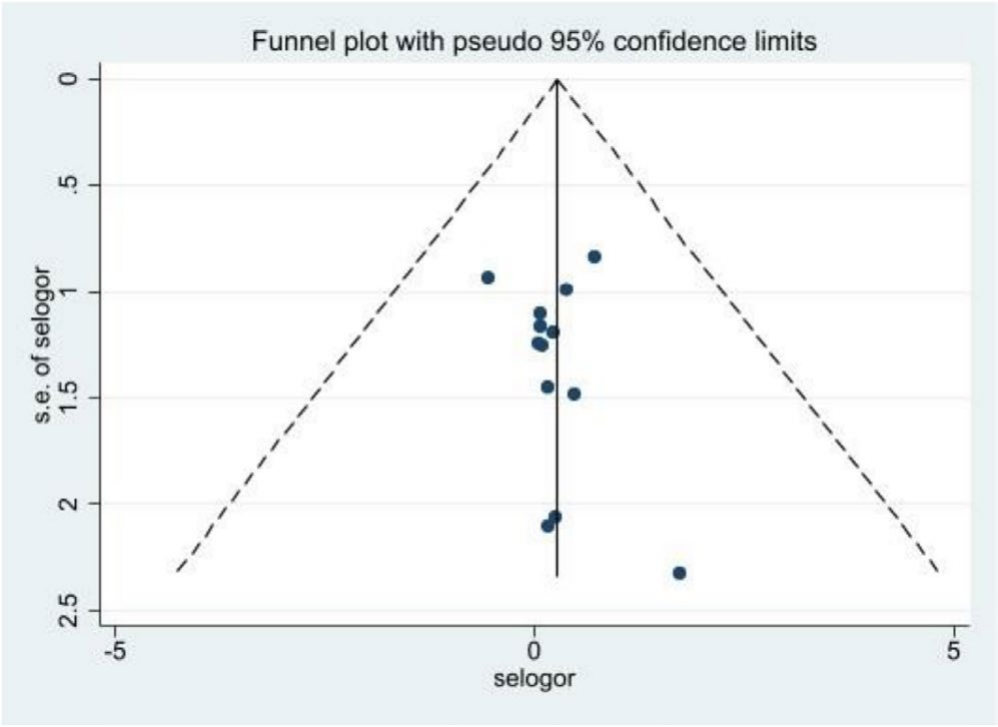
Bias publication assessment in the funnel plot.

The evaluation results showed that the publication bias did not negatively affect the overall association of SES inequalities with DM comorbidities in patients with diabetes, which is summarised in the distribution of these studies in the funnel plot. Accordingly, based on the results of Egger's test, there was no evidence of publication bias in the study results (Egger's test *t* = 0.18, 95% CI: −0.74, 0.37, *p*: 0.48) (Figure 5).

## 4 Discussion

For the first time, we examined the relationship between SES inequalities and T2DM comorbidity in a systematic review and meta‐analysis of 757,599 DM patients with a median age of 65 years. Out of every 100 patients with DM, 77 patients had at least one comorbidity. The most common comorbidities included hypertension, blood lipids and cardiac complications and outcomes.

The pooled estimate of 13 studies showed that the probability of DM comorbidities was significantly related to SES inequalities. The probability of DM comorbidities decreased significantly with increasing SES levels. The probability of DM comorbidities was 1.3 times higher in DM patients with low SES than in DM patients with high/middle SES. Gender subgroup analysis showed that the probability of DM comorbidities was higher in women than in men.

To control the significant heterogeneity between studies, meta‐regression analysis was performed. The results of the meta‐regression showed that the prevalence of comorbidity (per 10%), mean age, education years, Quality of studies, sample size and geographic region were significantly associated with the overall effect. A significant part of this heterogeneity between studies can be explained by differences in the characteristics of the patients studied in the included studies (age distribution, sex, race, etc.), differences in geographical areas (access to screening, diagnostic and treatment services, including access to antidiabetic drugs and screening for other comorbidities) and differences in the definition of SES and study design. Studies have shown that the prevalence of comorbidities is higher in women than in men [8, 37, 38]. Christiani et al. [39] showed in a systematic review that in diabetic patients, low SES was significantly associated with a lack of access to antidiabetic drugs. Although we identified factors associated with heterogeneity between studies through meta‐regression analysis, it is possible that a part of the remaining heterogeneity between studies is due to differences in the SES measurement indicators in different studies, so this should be taken into account when interpreting the results, although the majority of studies used a combination of income, occupation and literacy elements.

In a systematic review, Tatulashvili et al. [6] examined the relationship between SES inequalities and DM complications, including retinopathy and cardiopathy, and showed that low SES was significantly associated with an increased risk of diabetes‐related complications. In a systematic review, Scott et al. [40] examined the effect of socioeconomic inequalities on mortality, morbidity and management of diabetes in patients with type 1 diabetes. They showed that mortality and complications of type 1 diabetes were significantly higher in patients with low SES, which may be related to poor disease management due to low SES. In another systematic review, Grintsova et al. [10] examined healthcare disparities among patients with T2DM based on individual SES and regional deprivation. Despite differences in research approaches, they showed that health care (both on the process of care and intermediate outcome indicators) was significantly poorer in patients with low SES. They reported that the available evidence indicated the existence of significant socioeconomic disparities in diabetes care. Individuals with low SES and those living in deprived areas are frequently linked to poorer process measures and inferior overall results, which in turn lead to increased risks of both microvascular and macrovascular complications.

In a systematic review, Peykari et al. [8] by evaluating 15 related articles showed that SES inequality was significantly related to the increase in the prevalence of diabetes. The prevalence of diabetes was higher in people with lower SES, especially in women. Also, in their study, diabetes complications were more common in patients of higher age and lower income status. They reported that socioeconomic factors were associated with diabetes that may lead to inequalities in access to healthcare services. In our study, we showed that in addition to diabetes itself, SES inequalities were also related to an increase in DM comorbidity, which one of the main reasons can be due to the inequality of diabetic patients in access to healthcare services, including access to antidiabetic drugs and screening for other comorbidities and the susceptibility of people with low SES to other diseases, especially in women [41, 42]. In a systematic review, Humphries et al. [43] showed that, although most of the risk factors of heart diseases are common in women and men, the risk of developing cardiovascular diseases was higher in women than in men.

The results of this study further highlight the need for health policymakers to pay attention to patients with T2DM. Given the high prevalence of type 2 diabetes and its associated comorbidities in individuals and regions with low SES, health policymakers should pay significant attention to this population group and reduce the burden of these comorbidities in this group by designing and implementing screening programs and providing free access to diagnostic and treatment services. Designing prospective studies to assess the role of socioeconomic inequality in the development of comorbidities with other common noncommunicable diseases, including hypertension, cardiovascular diseases and obesity, could help to more accurately estimate the role of socioeconomic factors on the burden of disease.

### 4.1 Limitations

Our study was about the strengths and weaknesses that should be pointed out. First, due to the design of the primary studies included in this systematic review, we could not assess the association of SES inequalities with DM comorbidities in diabetic patients based on several important variables: the number of comorbidities, age and education level. Second, another important limitation of this systematic review was the differences in SES measurement indicators across studies, which may have affected the study results, although the majority of studies used a combination of income, occupation and literacy elements. Third, most primary studies were conducted in developed countries, and their results should be cautiously generalised to other countries. Fourthly, the studies conducted in this meta‐analysis were conducted in different study environments, periods, places and populations, which can affect the study's results to some extent. Examining the relationship between SES inequalities and DM comorbidities for the first time in a meta‐analysis was the most important strength of this study.

## 5 Conclusion

SES inequalities as a dependent factor were significantly associated with DM comorbidities. The potential of DM comorbidities may be higher in T2DM patients at the lowest SES than in patients with middle and higher SES. The potential of DM comorbidities was higher in women compared to men and in patients with low income compared to high. Improving healthcare systems and interventions to reduce SES inequalities in T2DM patients are recommended. Healthcare systems are expected to highlight gaps in preventive and care services provided to the most vulnerable.

## Author Contributions

A.G., M.B. and S.S.: formulated the initial research question, data analysis and data acquisition. A.S., M.B. and F.M.: data analysis/interpretation. M.B., F.M.T. and A.G.: critically reviewed and provided substantial revisions to the manuscript. A.G.: supervision and mentorship.

## Funding

The authors have nothing to report.

## Ethics Statement

Protocol registered in PROSPERO (registration number: CRD42024604213).

## Conflicts of Interest

The authors declare no conflicts of interest.

## Supporting information


**Data S1:** edm270160‐sup‐0001‐Supinfo1.docx.

## Data Availability

The data that support the findings of this study are available on request from the corresponding author. The data are not publicly available due to privacy or ethical restrictions.
