# Supplementary material for: Socioeconomic Inequalities and Type 2 Diabetes Comorbidities: A Systematic Review and Meta‐Analysis on Observational Studies
Source: Endocrinol Diabetes Metab. 2026 Jan 22;9(1):e70160. doi: 10.1002/edm2.70160 (PMC12828065; doi:10.1002/edm2.70160)
Supplement: Supplementary file 1 — Data S1: edm270160‐sup‐0001‐Supinfo1.docx. [file EDM2-9-e70160-s001.docx]

1. General search strategy for searching for resources

| general search strategy |
| --- |
| ( " Diabetes Mellitus, Type 2 " OR " Adult-Onset Diabetes Mellitus " OR " Diabetes Mellitus " OR " Stable Diabetes Mellitus " OR’’ Type 2 Diabetes " OR " Diabetes Mellitus, Noninsulin-Dependent " OR " Diabetes ’’) AND ( " Socioeconomic Status " OR ‘’ Socioeconomic Class’’ OR " Socioeconomic Level " OR " Socioeconomic Factor " OR " Socioeconomic inequalities " OR " Socioeconomic Characteristic " OR '' Socioeconomic deprivation'' OR "neighborhood marginalization " OR " geographical inequalities " OR " Socioeconomic inequalities " OR " socioeconomic disparity’’" OR ‘’ Poverty’’ OR " Educational Status " OR " Income " OR " Occupations " OR " socioeconomic disparity )AND ( " Comorbidity" OR ‘’ Multimorbidity’’ OR " microvascular complications’’ OR " Noncommunicable Diseases" OR ‘’ Noninfectious Diseases " OR ‘’ Non communicable Diseases’’ OR ‘’ Non-communicable Chronic Disease’ ’OR " Macrovascular outcomes’’) |

Table S1: Assessing the quality of studies based on the checklist for assessing the quality of non-randomized studies

| **Author, Year, reference** | **Selection** | **Comparability** | **Outcome** | **Classification based on AHRQ standards** |
| --- | --- | --- | --- | --- |
| R Dray-Spira  (2008)[29] | ** | * | ** | Moderate |
| V Fano  (2013)[25] | **** | ** | *** | High |
| PC Chen  (2015)[30] | **** | ** | *** | High |
| J Walker  (2016)[23] | **** | ** | ** | High |
| B Ibáñez  (2018)[12] | **** | ** | *** | High |
| N Ali  (2019)[31] | *** | * | ** | Moderate |
| T Biswas  (2019)[24] | **** | ** | *** | High |
| L Bartolini  (2020)[32] | **** | ** | *** | High |
| P Jiang  (2020)[33] | *** | ** | ** | High |
| AC Falkentoft  (2021)[34] | **** | ** | *** | High |
| S Kundu(2022)  [35] | ** | * | ** | Moderate |
| B Safieddine  (2023)[14] | *** | ** | *** | High |
| J Uddin(2023)  [22] | **** | ** | *** | High |


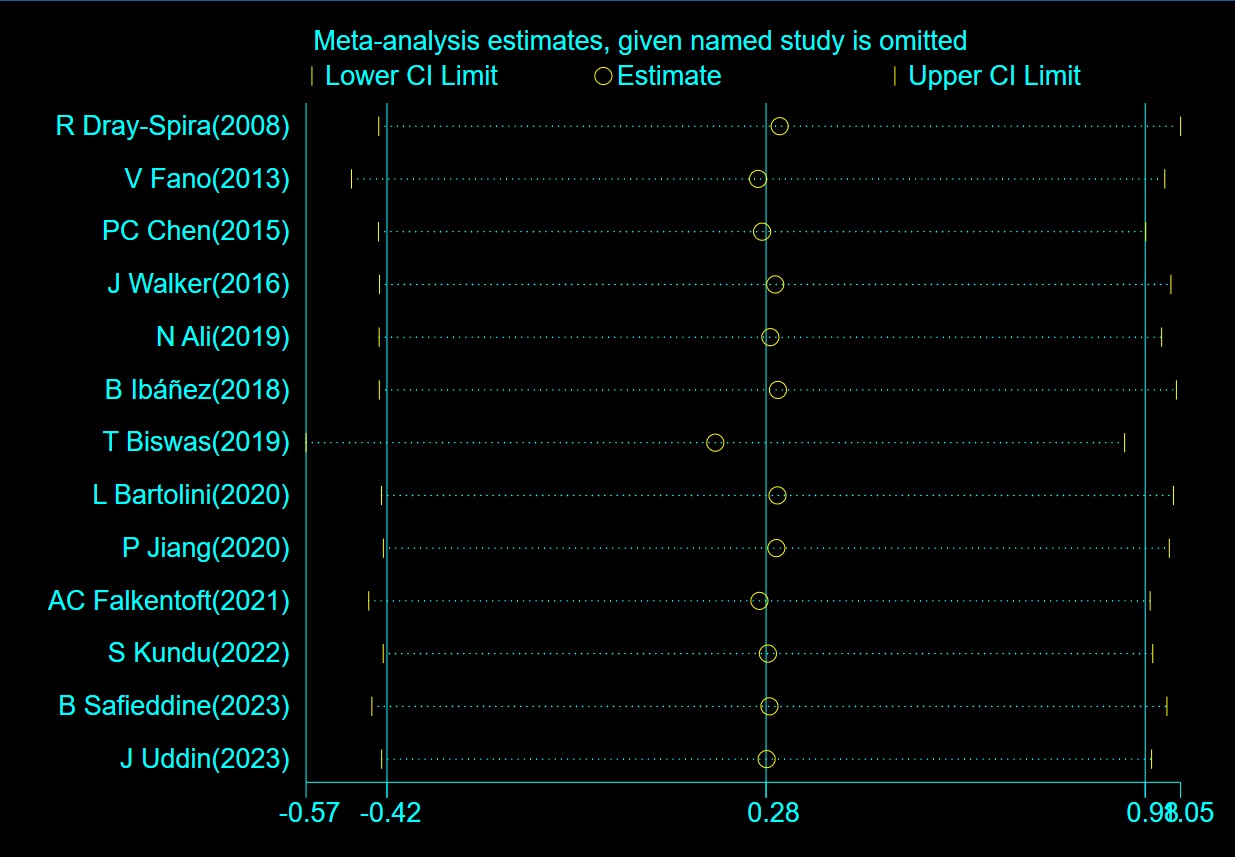


Figure S1: Sensitivity analysis results
